# Stochastic surprisal: An inferential measurement of free energy in neural networks

**DOI:** 10.3389/fnins.2023.926418

**Published:** 2023-03-14

**Authors:** Mohit Prabhushankar, Ghassan AlRegib

**Affiliations:** Omni Lab for Intelligent Visual Engineering and Science (OLIVES), Georgia Institute of Technology, Electrical and Computer Engineering, Atlanta, GA, United States

**Keywords:** free-energy principle, neural networks, stochastic surprisal, image quality assessment, robust recognition, human visual saliency, abductive reasoning, active inference

## Abstract

This paper conjectures and validates a framework that allows for action during inference in supervised neural networks. Supervised neural networks are constructed with the objective to maximize their performance metric in any given task. This is done by reducing free energy and its associated surprisal during training. However, the bottom-up inference nature of supervised networks is a passive process that renders them fallible to noise. In this paper, we provide a thorough background of supervised neural networks, both generative and discriminative, and discuss their functionality from the perspective of free energy principle. We then provide a framework for introducing action during inference. We introduce a new measurement called stochastic surprisal that is a function of the network, the input, and any possible action. This action can be any one of the outputs that the neural network has learnt, thereby lending *stochasticity* to the measurement. Stochastic surprisal is validated on two applications: Image Quality Assessment and Recognition under noisy conditions. We show that, while noise characteristics are ignored to make robust recognition, they are analyzed to estimate image quality scores. We apply stochastic surprisal on two applications, three datasets, and as a plug-in on 12 networks. In all, it provides a statistically significant increase among all measures. We conclude by discussing the implications of the proposed stochastic surprisal in other areas of cognitive psychology including expectancy-mismatch and abductive reasoning.

## 1. Introduction

The human visual system is the resultant of an evolutionary process influenced and constrained by the natural visual stimuli present in the outside environment (Geisler, 2008; Sebastian et al., 2017). The free energy principle is an over-arching theory that provides a mathematical framework for this evolutionary process (Friston, 2009). The principle provides a theory of cognition that can unify and discuss relationships among fundamental psychological concepts such as memory, attention, value, reinforcement, and salience (Friston, 2009). It decomposes the visual system into perception and action modalities and argues that the visual system is an inference engine whose objective is to perceive the outside environment as best as it can. If this perception is insufficient for making an inference, an action is taken to achieve the objective by influencing the outside environment. While the action is dependent on the type of inference that is to be made, perception is dependent on the natural visual stimuli. Hence, a study of the human visual system warrants a study of the patterns that it is sensitive to. Broadly, these patterns are classified under natural scene statistics (Geisler, 2008). Color, luminance, spatio-temporal structures and spectral residues are some statistics that are useful in performing fundamental visual tasks including image quality assessment (Zhang and Li, 2012), visual saliency detection (Hou and Zhang, 2007), and object detection and recognition (Sebastian et al., 2017).

Image quality assessment is the objective assessment of subjective quality of images. Visual saliency detection finds those regions in an image that attract significant human attention. Object recognition attempts to recognize any given object in an image. Methods like Hou and Zhang (2007) and Murray et al. (2013) use spectral residue to detect salient regions. Hou and Zhang (2007) extend their spectral residue-based saliency detection algorithm to show that object detection is possible. The spectral residual concept is used in SR-SIM (Zhang and Li, 2012) and BleSS (Temel and AlRegib, 2016a) to utilize the frequency characteristics to quantify residuals for IQA. All three disparate applications share commonalities in their spectral residual statistics that are used to show comparable performance within each application. Hence, natural scene statistics and their governing visual system principles are building blocks of computational machine vision systems that attempt to mimic human perception.

One such a principle is the consistency in spatial structures that allows for a sparse set of convolutional kernels to represent natural scenes. Large-scale neural networks are built on this principle. Neural networks are empowered to mimic human vision by performing the same tasks as the human visual system including image quality assessment (Temel et al., 2016), visual saliency detection (Sun et al., 2020), and object recognition (Krizhevsky et al., 2012) among others. Recently the generalization capabilities of neural networks has led to their widespread adoption in a number of computational fields. Neural networks have produced state-of-the-art results on multifarious data ranging from natural images (Krizhevsky et al., 2012), computed seismic (Shafiq M. A. et al., 2018; Shafiq M. et al., 2018), and biomedical images (Prabhushankar and AlRegib, 2021b; Prabhushankar et al., 2022). In object recognition on Imagenet dataset (Deng et al., 2009), He et al. (2016) surpassed the top five human accuracy of 94.9%. In the application of image quality assessment, Bosse et al. (2017) extracted patch-wise distortion characteristics from images using deep neural networks before fusing them to obtain an objective quality score. The authors in Liu et al. (2017) device a sparse representation-based entropic measure of quality that is inspired by the free energy principle. This is extended in Liu et al. (2019) where the authors use the free energy principle as a plug-in on top of existing blind image quality assessment techniques. In both these works, free energy principle is seen as a technique that measures the disparity between an outside environment and the expectation of that environment through some biologically plausible mechanism. Other existing works, including Zhai et al. (2011) and Gu et al. (2014), quantify this disparity to estimate quality.

Hence, from the perspective of free energy principle, neural networks act as biologically plausible mechanisms to perceive the outside environment. This is done by supervising the networks to learn particular tasks. Prabhushankar and AlRegib (2021a) describe supervised learning as associative learning where a set of learned features is associated with any given class. This class can be an objective score in image quality assessment or an object class from recognition. The learned features are associated with a specific dataset and application, and are not easily transferable (Temel et al., 2018). A number of recent works including (Goodfellow et al., 2014; Temel et al., 2017; Hendrycks and Dietterich, 2019) describe the fallibility of neural networks to adversarial noise and slight perturbations in data arising from acquisition or environmental errors. The feature representation space can be altered significantly by noise that is sometimes non-noticeable in data. This is in contrast with the spectral residual feature which is used to infer both object (Hou and Zhang, 2007) and image quality (Zhang and Li, 2012; Temel and AlRegib, 2016a).

We posit that these shortcomings of supervised neural networks are a resultant of neural networks exclusively utilizing the perception modality of free energy principle. In other words, the passivity of neural networks during inference leads to their non-robust nature. This view is corroborated by Demekas et al. (2020) who identify three challenges in supervised learning. Firstly, they claim that neural networks lack an explicit control mechanism of incorporating prior beliefs into predictions. Secondly, neural networks train *via* a scalar loss function that does not allow for incorporating uncertainty in action. Lastly, neural networks do not perform any action during inference that would elicit changes in the input from the outside environment.

In this paper, we tackle the above challenges by introducing a framework for action during inference. This is opposed to the free energy principle based works in Liu et al. (2017, 2019) where the methodology does not require actions at inference. Based on the free energy principle, we treat any trained neural network as an inference engine. We define a quantity called *stochastic surprisal* that is a function of a neural network's inference and some action performed on this inference. Reducing surprisal is generally seen as a single action that reduces the distributional difference between two quantities. However, during inference, we have access to only a single data point. We overcome this challenge by considering that all possible actions that the network can undertake are equally likely. The term *stochastic* is derived based on this assumption of action-randomness. Stochastic surprisal acts on top of *any* existing neural networks to address the challenge of passive inference. Existing neural networks can either be generative or discriminative. We evaluate stochastic surprisal on two applications including image quality assessment and robust object recognition. In image quality assessment, we evaluate our technique to assess the quality of distorted images at different levels of distortions. Similarly, in robust object recognition, we recognize distorted images when the original neural network is only trained on pristine images. In other words, we propose a concept that is able to assess the noise characteristics in images to assign objective quality, as well as ignore the same noise characteristics to robustly classify images. The contributions of this paper include,

- We unify the concepts of image quality assessment and robust object recognition. We show that the features that are extracted from neural networks simultaneously characterize the scene and context within the image for recognition as well as the noise perturbing it's quality.
- We term our features as *stochastic surprisal* and relate them to the free energy principle. We provide a mathematical framework to extract stochastic surprisal from both discriminative and generative neural networks as a function of some action.
- We discuss the implications of our proposed method from an abductive reasoning as well as expectancy-mismatch perspective. Both these concepts lead to separate applications including context and relevance based contrastive visual explanations and human visual saliency detection.

We first describe the free energy principle in Section 2.1.1. The free energy principle is then related to neural networks in Section 2.1.2 before describing stochastic surprisal. The generation of stochastic surprisal in generative and discriminative networks is described in Sections 2.1.2.1 and 2.1.2.2, respectively. Finally, the applications of image quality assessment and robust recognition and our methodology is discussed in Section 2.3. The results are provided in Section 3. We further discuss the implications of the proposed stochastic surprisal on other cognitive concepts and conclude in Section 4.

## 2. Theoretical overview and methodology

In this section, we provide a thorough background of the free energy principle and its application in neural networks, both generative and discriminative. We then define and detail the framework for the extraction of stochastic surprisal. This is followed by the application of stochastic surprisal in image quality assessment and robust recognition.

### 2.1. Background

#### 2.1.1. Free energy principle

The Free Energy Principle (FEP) proposes a theory to explain the self-organizing capability of any intelligent and adaptive system (Friston, 2009). FEP assumes the demarcation of a *system* that exists in an *environment* through a functional *Markov Blanket*. The Markov Blanket (Hipólito et al., 2021) provides statistical independence to the system from its environment, thereby imbuing the system with a sense of *self* . A consequence of this separation is that the system only experiences the environment through the Markov Blanket based on a limited set of sensory inputs. These sensory inputs are used to create a generative model of the outside environment within the system. The system then performs a limited set of actions affecting the outside environment while updating its internal model of the outside environment. The FEP provides a mathematically concrete set of principles to bound the long-term entropy of the internal generative model that is confined in the set of all possible sensory inputs and its possible performative actions. Friston (2019) argues that the assumption of the Markov Blanket and the ensuing FEP is an overarching theory that provides a tool to study and explain self-organization at any spatio-temporal scale from infinitesimal quantum mechanics to generational biological evolution.

In this paper, we are interested in the FEP's application to visual processes related to the human brain. The applicability of FEP across concepts such as memory, attention, value, and reinforcement (Friston, 2009) is possible because of the central assumption that the *limited* sensory inputs from the outside environment to the brain are also *likely* sensory inputs. In other words, the human brain only allows for a *limited* set of *likely* encounters (Demekas et al., 2020). The term *likely* is a function of the expectation set by the internal generative model within the brain. Hence, the brain is considered to encode a Bayesian recognition density that predicts the sensory inputs based on some hypothesis regarding their cause. This leads to the proposition that the brain is an inverse generative model where it expects to sense only a limited set of likely inputs from the environment. Any mismatch to this expectation is handled in two stages. Firstly, the internal model is updated with the mismatched sensory input to improve the *perception*. Secondly, an action is performed to change the environment. This way, the environment and the model are made to fit each other by reducing the mismatched input. A mismatched input is typically termed as a *surprising* event (Buckley et al., 2017). Self-organization in the brain creates an imperative to minimize the *surprisal* of any event and the FEP provides a mathematical theory of this minimization by providing a tractable upper bound to the surprisal. Mathematically, average surprisal is the entropy of the distribution of all events. More *unlikely* an event, more *surprisal* it creates in the internal model. The free energy decomposed using surprisal (Demekas et al., 2020) is given by,


(1) $$\begin{array}{l} \text{Free Energy}=\text{Divergence}+\text{Surprisal}. \end{array}$$

Here, divergence is the difference between the variables representing the outside environment that generate the sensory inputs and the variables in the internal generative model that mimic the outside world.

#### 2.1.2. Free energy principle in neural networks

The assumption of the existence of an internal tractable generative model that is an inference engine has been adopted in the construction of early neural networks. Hinton and Zemel (1993) describe the Helmholtz free energy that is used to construct autoencoders as agents that minimize the reconstruction cost and the code cost. The code cost is a function of the entropy of the probability distribution given a vector. In FEP, this code cost is the surprisal. Variational Autoencoders (Kingma and Welling, 2019) minimize Variational Free Energy (VFE) and consequently surprisal. VFE is a generalization of the Helmholtz free energy where the divergence of the approximate and true probabilities are minimized (Gottwald and Braun, 2020). While the generative models of autoencoders lend themselves directly to the FEP, the discriminative models also train themselves using some variation of a loss function that resembles free energy. In this paper, we use both generative and discriminative models and we introduce them in terms of the free energy principle.

##### 2.1.2.1. Generative networks

In this section, we consider a general autoencoder as our generative model. An autoencoder is an unsupervised learning network which learns a regularized representation of inputs to reconstruct them as its output (Hinton and Zemel, 1993; Kwon et al., 2019). Since Hinton and Zemel (1993), a number of variations have been proposed to autoencoders to construct either application-specific or property-specific networks. These variations generally deal with constraining the latent representations learned by an autoencoder. For instance, Ng (2011) constrain the latent representation to be sparse, thereby constructing sparse autoencoders. Kingma and Welling (2013) constrain the latent representation to follow a Gaussian distribution. These are termed as variational autoencoders. These are two instances of property-specific autoencoders. Application-specific autoencoders include fully-connected networks used for image compression (Gedeon and Harris, 1992), and convolutional autoencoders for image denoising (Mao et al., 2016).

**Figure 1:**
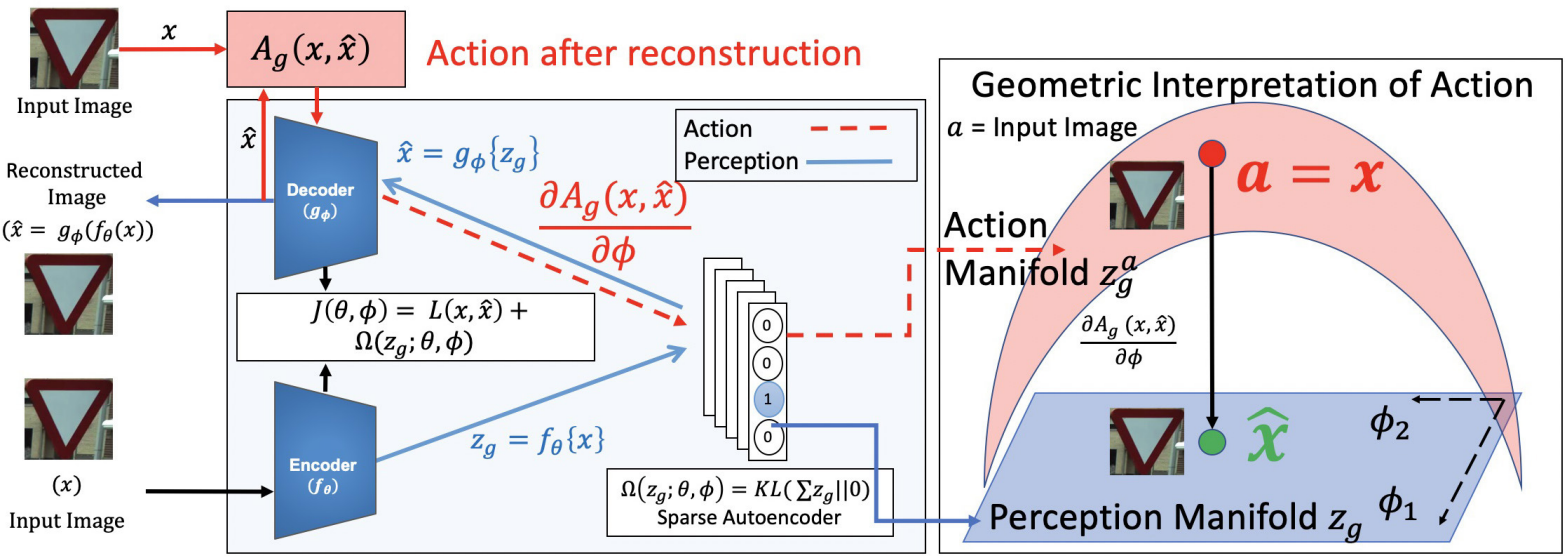
Block diagram for perception (in blue) and proposed action (in red) for a sparse autoencoder. The image *x* is taken from the CURE-TSR dataset (Temel et al., 2017). The training loss function is *J*(θ, ϕ). The latent representation *z* = *f*_θ_(·) is *z*_*g*_. The reconstructed image is shown as $\hat{x}$. This forms the perception pipeline. The action pipeline is shown in red where the action $A_{g}$ is backpropagated through the decoder to the latent representation space. The learned blue perception representation space *z*_*g*_ changes to the action space $z_{g}^{a}$ as a consequence of $A_{g}$. This change is stochastic surprisal, given by $\frac{\partial A_{g}\left(x,\hat{x}\right)}{\partial \phi }$.

All these autoencoders consist of the same base architecture as shown in Figure 1. They consist of an encoder *f*_θ_(·), parameterized by θ to map inputs *x* to a latent representation *z*_*g*_. These latent representations *z*_*g*_ are used to reconstruct the same input $\hat{x}$ using a decoder *g*_ϕ_(·). This operation is mathematically represented as,


(2) $$\begin{array}{l} z=f_{\theta }\left(x\right)\;\hat{x}=g_{\phi }\left(z\right)=g_{\phi }\left(f_{\theta }\left(x\right)\right), \end{array}$$

For a natural image input, *x* ∈ ℝ^*H* × *W* × *C*^, where *H, W, C* are height, width, channel of input image, respectively. The encoder and decoder are trained jointly by minimizing a loss function *J*(θ, ϕ) defined as:


(3) $$\begin{array}{l} J\left(\theta ,\phi \right)=L\left(x,g_{\phi }\left(f_{\theta }\left(x\right)\right)\right)+\Omega \left(z_{g};\theta ,\phi \right), \end{array}$$

where L is a reconstruction error which measures the dissimilarity between the input *x*, and the reconstructed image $\hat{x}$. Ω is a regularization term added to avoid overfitting the network to the training set and to imbue the required constraints. For a sparse autoencoder, Ω is an *l*_1_ sparsity constraint. However, since the *l*_1_ constraint is not differentiable, a practical solution for constructing this sparsity constraint is to use KL-Divergence on *z*_*g*_. Specifically, the sum of *z*_*g*_ is constrained to either zero or a very small value using a distance metric like KL-Divergence. This is shown in Figure 1 in blue.

During training, the network parameters, θ and ϕ are updated by backpropagating the gradients of *J*(θ, ϕ) w.r.t. the parameters. The update rule is given by,


(4) $$\begin{array}{l} \theta^{\prime }=\theta -\frac{\partial J\left(\theta ,\phi \right)}{\partial \theta },\;\phi^{\prime }=\phi -\frac{\partial J\left(\theta ,\phi \right)}{\partial \phi }, \end{array}$$

The two gradients provide the change in the network parameters required to incorporate better perception capabilities as measured by the loss function *J*(θ, ϕ).

Consider Equation (3) and compare this against the free energy decomposition in Equation (1). The L reconstruction error measures the divergence. The regularization is the surprisal. Technically, regularization prevents the network from reconstructing *x* exactly. Hence, surprisal is *added* in generative networks to make them generalizable. A thorough analysis of regularization for reconstruction and feature transfer of autoencoders to multiple tasks is provided in Prabhushankar et al. (2018). While regularization impacts the reconstruction negatively, it enhances the adaptability and usability of features for generalized tasks and test sets.

##### 2.1.2.2. Discriminative networks

**Figure 2:**
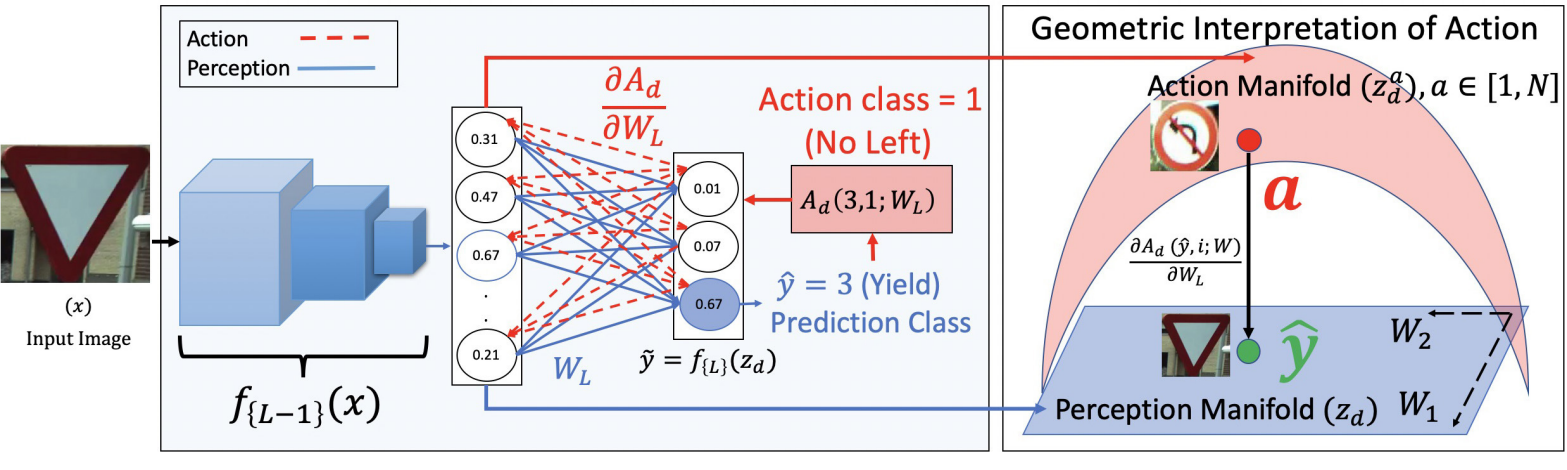
Block diagram for perception (in blue) and proposed action (in red) for a classification network. The image *x* is taken from the CURE-TSR dataset (Temel et al., 2017). The perception pipeline is shown in blue where the network assigns a class 3 to *x*. The action pipeline is shown in red where the action $A_{d},a=1$ is backpropagated through the final fully connected layer to the learned blue perception manifold *z*_*d*_. *z*_*d*_ changes to the action manifold $z_{d}^{a}$ as a consequence of $A_{d}$. This change is stochastic surprisal, given by $\frac{\partial A_{d}\left(ŷ,i;W\right)}{\partial W_{L}}$.

Discriminative networks are neural networks whose function is to assign labels to input data. While the required training data in generative networks are images *x* ∈ ℝ^*H* × *W* × *C*^, the training data for discriminative networks are (*x, y*), where *x* ∈ ℝ^*H* × *W* × *C*^ and *y* ∈ [1, *N*]. Here, *y* is an integer label assigned to *x*, ranging between 1 and the total number of classes *N*. The goal of a discriminative network is to assign the label *y*, given *x* at inference. The simplest discriminative network is an image classification network. Consider an *L*-layered network *f*(·) trained to classify images on a domain X. For the task of classification, where *f*(·) is trained to classify between *N* classes, the last layer is commonly a fully connected layer consisting of *N* weights or filters. During inference, the representation space *z*_*d*_ = *f*_*L*−1_(*x*) after the first (*L*−1) layers are projected independently onto each of the *N* filters. The filter with the maximum projection is inferred as the class ŷ to which *x* belongs. Mathematically, *z*_*d*_ and ŷ are related as,


(5) $$\begin{array}{l} z_{d}=f_{L-1}\left(x\right), \end{array}$$

(6) $$\begin{array}{ll} ỹ=\mathrm{argmax} & \left(W_{L}^{T}z_{d}+b_{L}\right),\;ŷ=\mathrm{argmax}\left(ỹ\right) \end{array}$$

(7) $$\begin{array}{ll} \forall W_{L}\in {ℜ}^{d_{L-1}\times N}, & \;z\in {ℜ}^{d_{L-1}\times 1},b_{L}\in {ℜ}^{N\times 1},ỹ\in {ℜ}^{N\times 1},ŷ\in \left[1,N\right], \end{array}$$

where *W*_*L*_ and *b*_*L*_ are the parameters of the final fully connected layer. Note our choice of the variable *z*_*d*_. This is a similar variable that is used to denote the latent representation in Equation (2). Similar to the decoder *g*_ϕ_(·) acting on *z*_*g*_ in generative networks, we have the final fully connected layer *W*_*L*_ and *b*_*L*_ acting on *z*_*d*_. This forms the perception pipeline that classifies *x* as ŷ. This is shown in blue in Figure 2.

Training an image classification technique requires a loss function *J*(ŷ, *y*; θ), where θ are the network parameters and (*x, y*) are the image-label pairs required for training. A common choice of *J*(·) is the cross-entropy loss. Considering σ(ỹ) to be the softmax probability distribution of the output vector from *f*(·), the cross-entropy loss interms of KL-Divergence and entropy can be expressed as,


(8) $$\begin{array}{l} J\left(\cdot \right)=\text{KL}\left(y||\sigma \left(ỹ\right)\right)-\overset{N}{\underset{i=1}{\sum }}\left(\sigma \left(\tilde{y_{i}}\right)\right)ln\left(\sigma \left(\tilde{y_{i}}\right)\right). \end{array}$$

Here, KL(||) refers to the KL-divergence between the probability output of the network and the label vector *y* expressed as a one-hot probability distribution. Notice the similarity between Equations (1) and (8). The divergence in the FEP is the KL divergence and the surprisal is the entropy given by the second term in Equation (8). Unlike the generative networks, surprisal is not introduced into the network. Rather, the existing surprisal is minimized. A number of foundational works in FEP (Friston, 2009, 2019) use the entropy of a distribution to describe free energy. The network is then trained by backpropagating the errors w.r.t θ similar to Equation (4).

##### 2.1.2.3. Terminologies

Before describing our contributions, we summarize a few key terminologies that are extensively used within the FEP setup and how they relate to neural networks.

###### 2.1.2.3.1. External state of the world

X is the observed distribution of the outside world and each x∈X is an instance of this distribution. When describing discriminative systems, data is denoted as (*x, y*) where *x* is the data point and *y* is its label. When dealing with generative models, data is *x* only. When there is some distortion associated with the outside environment, the sampled data is *x*′ and the distribution is $X^{\prime }$. We will see $X^{\prime }$ and *x*′ in IQA and recognition experiments when input data are distorted by noise.

###### 2.1.2.3.2. System

A neural network *f*(·) trained on a distribution X. A trained system is one that does not take in any external inputs to change or update its weights. We consider that a trained system is at NESS density. For a discriminative network, *f*(·) is the entire system and its training data is denoted by (*x, y*). For a generative network, *f*_θ_(·) is an encoder trained to produce a latent representation space *z*_*g*_ given data denoted by *x* and *g*_ϕ_(·) is the decoder trained to reconstruct the image given a latent representation *z*_*g*_.

###### 2.1.2.3.3. Markov blanket

The part of the system that produces the latent representation *z*. In a generative system the Markov blanket is the encoder *f*_θ_(·) and in a discriminative system, the Markov blanket is the initial part of the network from Equation (5), *f*_*L*−1_(·).

###### 2.1.2.3.4. Internal state of the system

Let *z* denote the internal state of the latent representation within a system. Given a generative network, the latent representation after the encoder, *z*_*g*_ = *f*_θ_(*x*) is the internal state. Given a discriminative network, the internal state is *z*_*d*_ = *f*_*L*−1_(*x*). The internal states of both the networks are interchangeably referred to as latent representations or as perception manifolds. Note that similar to external state, if an input *x* is distorted to *x*′, its internal state is also distorted and we will use either $z_{d}^{\prime }$ or $z_{g}^{\prime }$ to denote the internal state of the system. Given any action, *a*, the internal state shifts to *z*^*a*^ to accommodate this action without necessarily changing *x*. All these states are shown in Figures 1, 2.

### 2.2. Stochastic surprisal

During inference, the networks are passive. As discussed in Section 1 and noted by Demekas et al. (2020), there is no mechanism to include a non-scalar surprisal that allows for an action during inference. In this paper, we alleviate this challenge by defining a new quantity called *stochastic surprisal* as a function of a hypothetical action. Consider the differences in the existing definitions of surprisal. In generative networks from Equation (3), surprisal is the induced regularization that prevents overfitting and creates specific constraints for a latent representation *z*_*g*_. In discriminative networks from Equation (8), surprisal is the entropy of the network's predicted distribution obtained from a linear combination on *z*_*d*_. While the surprisal in Equation (1) deals with bounding the system's surprise of the distributional divergence between the internal model and external environment, the regularization-based and entropy-based definitions provide a mathematically-tractable definition in neural networks. In this paper, we provide a new mathematically-tractable definition of surprisal that is inherently a function of an action A and its effect on the network. A formal definition is provided first.

Definition 2.1 (Stochastic Surprisal). Given a trained neural network *f*_θ_(·) parameterized by θ, the gradient change $\frac{\partial A}{\partial \theta }$ with respect to the network parameters for all possible actions A from the perspective of *f*_θ_(·) is termed stochastic surprisal.

Stochastic surprisal measures the change required in the trained perception network to measure any given action A. It is stochastic since it does not measure the divergence between distributions but rather a single data point's influence on the network. It is a non-scalar value that acts on the network parameters according to Equation (4). Note that we do not actually update the network. Rather, we only measure the network update and use it as a surprisal quantity. This update is possible based on some action all of which are considered equally likely. A thorough discussion of the naming is provided in Section 4.1.

#### 2.2.1. Action and stochastic surprisal

Action is a function of any application. We first define it in a general fashion for generative and discriminative networks. In Section 2.3, we define it specifically for image quality assessment and robust recognition.

##### 2.2.1.1. Generative networks

The action in generative networks is straightforward. Given an image *x* and its reconstructed image $\hat{x}$, the possible action is to change the weight parameters in a way that reduces the disparity between *x* and $\hat{x}$. In this paper, we quantify this disparity as the Mean Square Error given by $∥x-\tilde{x}{∥}_{2}^{2}$. However, as described in Section 2.1.2.1, the surprisal is present in the regularization terms. Hence, any action performed has to account for this surprisal. In this paper, we use the elastic net regularization. The overall action that induces a change in the network is given by,


(9) $$\begin{array}{l} A_{g}=∥x-\hat{x}{∥}_{2}^{2}+\beta \overset{h}{\underset{j=1}{\sum }}\text{KL}\left(z_{j}||{\hat{\rho }}_{j}\right)+\lambda ∥W{∥}_{2}^{2}. \end{array}$$

where $A_{g}$ is a generative action. $∥x-\hat{x}{∥}_{2}^{2}$ is the MSE loss function, and $∥W{∥}_{2}^{2}$ is the regularization on the weights. $\overset{h}{\underset{j=1}{\sum }}\text{KL}\left(z_{j}||{\hat{\rho }}_{j}\right)$ is the sparsity constraint denoted as the divergence between the latent representation and some small value ${\hat{\rho }}_{j},j\in \left[1,h\right]$ where *h* is the size of the latent representation. By minimizing the KL divergence, the latent variables *z*_*j*_, *j* ∈ [1, *h*] are made sparse. β and λ are hyperparameters controlling the regularization.

Stochastic surprisal is the gradient of this action $A_{g}$ with respect to the decoder weights. The action pipeline along with the stochastic surprisal generation is shown in Figure 1 in red. At inference, a test image is passed through a trained network and reconstructed. The action from Equation (9) is calculated and backpropagated to the latent representation space *z*_*d*_. The change, measured as the gradients, creates a change in *z*_*d*_ and the new action manifold is termed $z_{d}^{a}$. A toy example of the geometric interpretation of this change is also shown Figure 1. The blue perception manifold *z*_*g*_ that reconstructs $\hat{x}$ is acted on by $A_{g}$ to obtain a new red action manifold $z_{d}^{a}$. The decoder can use this space to reconstruct *x* exactly. In Section 3, we show how these generated gradients can be used as features for image quality assessment. Note that we keep the perception pipeline as is and make no changes to the training process.

##### 2.2.1.2. Discriminative networks

The action $A_{d}$ in discriminative networks is more involved than generative networks. While in generative networks, the possible action is to reconstruct the image with higher fidelity, in discriminative networks, the action can take any one of *N* outcomes. At inference, discriminative networks are given an image *x* and asked to predict its label *y*. Assuming that ŷ is the prediction, the action we use to elicit change in the network parameters is by backpropagating an action class *a* in the loss function *J*(ŷ, *a*; *W*), *a* ∈ [1, *N*].


(10) $$\begin{array}{l} A_{d}=∥a_{i}-ỹ{∥}_{2}^{2},i\in \left[1,N\right]. \end{array}$$

Here *a*_*i*_ is the action class defined as a Kronecker delta function given by,


(11) $$\begin{array}{l} a_{i}=\{\begin{array}{cc} 1, & \text{if }i=\text{class},\\ 0, & \text{otherwise} \end{array} \end{array}$$

There is no regularization added to the discriminative action since the probability distribution σ(ỹ) derived from ỹ is a function of its surprisal entropy. Note that we use an MSE function for $A_{d}$ in Equation (10) similar to $A_{g}$ from Equation (9). An important difference between Equations (9) and (10) is the number of possible actions. In discriminative networks that classify between *N* classes, there are *N* possible *i* in Equation (10). Hence, there are *N* possible actions $A_{d}$ and *N* possible surprisals $\frac{\partial A_{d}^{i}}{\partial W_{L}},\forall i\in \left[1,N\right]$. The action pipeline for discriminative network for a toy example where the predicted class is 3 and the action class is 1 is shown in Figure 2 in red. The surprisals are the red gradients from the final fully connected layer. We also show the geometric interpretation of a given action on the learned representation space *z*_*d*_. The blue perception manifold is acted upon by $A_{d}^{1}$ through $\frac{\partial A_{d}^{1}}{\partial W_{L}}$ to obtain the red action manifold. Note that there are *N* such possible red $z_{d}^{a}$ due to the *N* possible actions. This idea of *N* separate gradients to characterize data is not new. In Settles et al. (2007), the authors construct positive and negative instance labels for a given input *x* in a binary decision setting. This is done to quantify uncertainty in an active learning setting. In this paper, we extend this characterization to *N*-label settings and use the image-label pairs to extract stochastic surprisal from the network.

Notice the difference in the definitions of action. In FEP, the generative model acts on the outside world creating a change that reduces its surprisal. Our definition in Equation (10) is the same one that is used in I-FGSM (Goodfellow et al., 2014) adversarial generation technique. Equation (10) is continuously applied and a gradient w.r.t. the input, i.e., $\frac{\partial A_{d}}{\partial x}$, is added to *x* until the prediction changes adversarially. Changing the input would be a true action from the FEP sense. However, in this paper, we do not explicitly change the outside world or *x*. Rather, we measure the effect of such a change on the network using $\frac{\partial A_{d}}{\partial W_{L}}$ without making said change.

### 2.3. Methodology

We validate the effectiveness of stochastic surprisal during inference on two applications: Image Quality Assessment (IQA) and Robust Classification. The action gradients, $\frac{\partial A}{\partial \phi }$ are used in two ways. The first approach is to use the surprisal gradients as error directions. This is done by projecting images with and without distortions onto the gradient space and comparing them. In this case, the surprisal acts as a measurement between the images and acts as a Full-Reference IQA metric. The second approach is to directly use surprisal gradients as feature vectors. The directional change caused by the actions is dependent on the network, the input and the action class. By keeping the network same across action classes, surprisal becomes a characteristic of the data. This approach is explored for the application of robust classification.

#### 2.3.1. Image quality assessment

Image quality assessment is a field of image processing that objectively estimates the perceptual quality of a degraded image. Multiple methods have been proposed to predict the subjective quality of images (Wang et al., 2003, 2004; Sampat et al., 2009; Ponomarenko et al., 2011; Wang and Li, 2011; Zhang et al., 2011; Mittal et al., 2012; Zhang and Li, 2012; Prabhushankar et al., 2017a, 2018; Temel and AlRegib, 2019). All these methods extract structure related hand-crafted features from both reference and distorted images and compare them to predict the quality. Recently, machine learning models directly extract features from images (Temel et al., 2016; Bosse et al., 2017; Prabhushankar et al., 2017b). The authors in Bosse et al. (2017) propose to do so in either the presence or absence of the original pristine image. In Ma et al. (2021), the authors propose a free energy inspired technique to predict the quality. They use a Generative-Adversarial Network as the base perception module and an additional CNN to model content and degradation dependent characteristics. In this paper, we approach the action module in FEP as a function of the perception module itself. We do so by extracting stochastic surprisal from the same perception network. Hence, our method acts as a plug-in on top of existing quality estimators. In this paper, we show quantitative results by plugging-in on top of UNIQUE (Temel et al., 2016) and qualitative results on top of Bosse et al. (2017). We first describe and motivate the usage of UNIQUE for quantitative results.

##### 2.3.1.1. UNIQUE

We choose UNIQUE as the base technique since it follows the generative process described in Section 2.1.2.1 and Figure 1. This allows for the generation of stochastic surprisal from Equation (3) based on the Action in Equation (9). The authors in Temel et al. (2016) train a sparse autoencoder with a one layer encoder and decoder and a sigmoid non-linearity on 100, 000 patches of size 8 × 8 × 3 extracted from ImageNet (Deng et al., 2009) testset. The autoencoder is trained with MSE reconstruction loss. This network is *f*(·) from Equation (3). UNIQUE follows a full reference IQA workflow which assumes access to both reference and distorted images while estimating quality. The reference and distorted images are converted to YGCr color space and converted to 8 × 8 × 3 patches. These patches are mean subtracted and ZCA whitened before being passed through the trained encoder. The activations of all reference patches in the latent space are extracted and concatenated. Activations lesser than a threshold of 0.025 are suppressed to 0. The choice of threshold 0.025 is made based on the sparsity coefficient used during training. Similar procedure is followed for distorted image patches. The suppressed and concatenated features of both the reference and distorted images are compared using Spearman correlation. The resultant is the estimated quality of the distorted image.

##### 2.3.1.2. Proposed methodology

**Figure 3:**
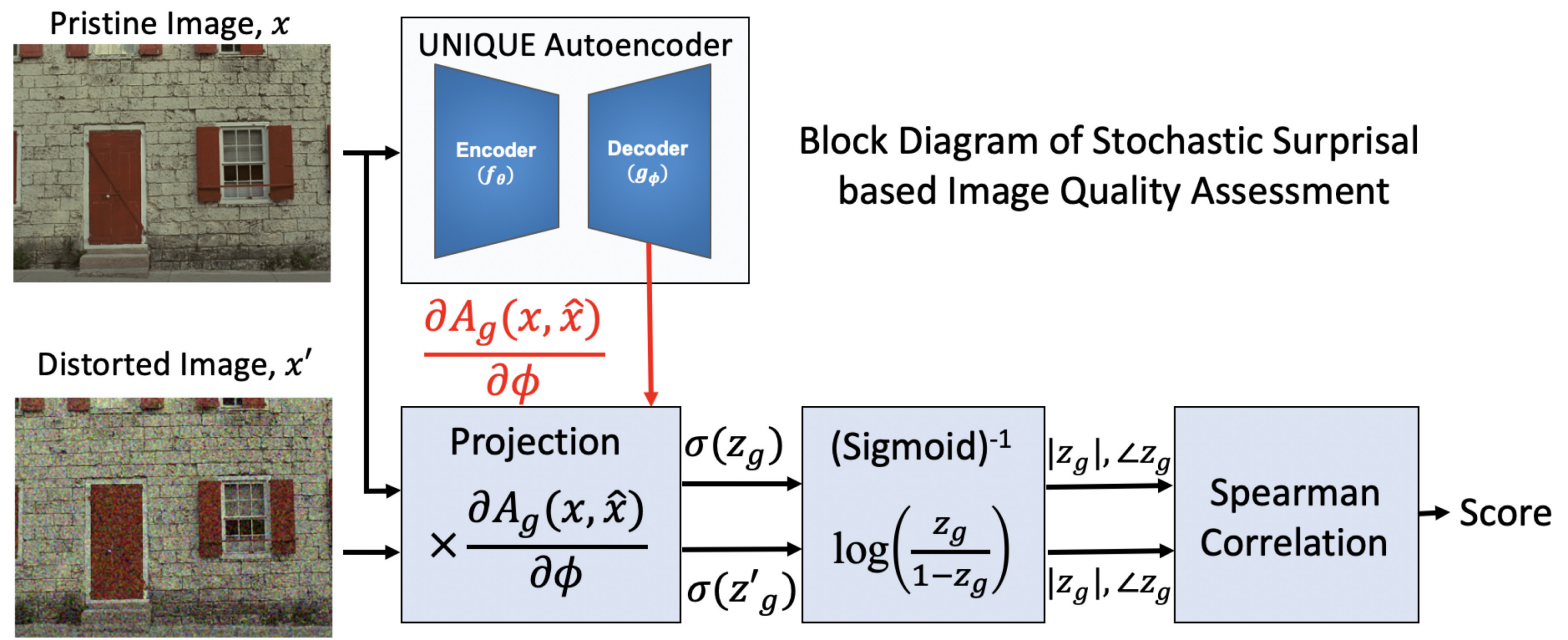
Block diagram of the proposed framework of IQA as a plug-in on top of Temel et al. (2016).

We provide the block diagram for the proposed methodology in Figure 3. Both the pristine and distorted images go through the same pre-processing steps detailed in UNIQUE (Temel et al., 2016) and are projected onto the stochastic surprisal gradients of the decoder. The gradients $\frac{\partial A_{g}}{\partial \phi }$ are extracted by backpropagating Equation (9). In this paper, we use the same hyperparameters β = 3, λ = 3*e*^−3^, and ρ_*j*_ = 0.035 as used in Temel et al. (2016). Once projected, the resultant is passed through an inverse sigmoidal layer to obtain the latent representation. Note that the latent representation is *z*_*g*_ for the pristine image and $z_{g}^{\prime }$ for the distorted image. Once passed through the inversion layer, both the magnitude and phase of each latent representation is concatenated and their spearman correlation coefficient is taken to estimate the quality score of the image.

#### 2.3.2. Robust classification

The goal is to characterize an image *x* using all *N* actions. Consider an image *x* whose class as predicted by *f*_θ_(·) is ŷ. Stochastic surprisal of *x* against class 1 is provided by backpropagating a loss between ŷ and 1 and obtaining corresponding gradients. The gradient is proportional to $A_{d}\left(ŷ,1;W_{L}\right)$, where *W* is the weight parameters and 1 is the action class. Specifically, it is $\nabla_{W_{L}}A_{d}\left(ŷ,1;W_{L}\right)$ for weights in layer *L* and class *i* ∈ [1, *N*]. We backpropagate over all *N* classes to obtain the overall surprisal features across all classes. The final feature, *r*_*x*_ for an image *x*, is given by concatenating all individual features and *r*_*x*_ is characteristic of image *x*. Hence,


(12) $$\begin{array}{l} \begin{array}{c} r_{i}=\left(\nabla_{W_{L}}A_{d}\left(ŷ,i;W_{L}\right)\right)),\forall i\in \left[1,N\right],\\ r_{x}=\left[r_{1},r_{2}\ldots r_{N}\right]. \end{array} \end{array}$$

Given a trained feed-forward network *f*(·) and image *x*, we extract gradients using Equation (12) which serve as our features. Gradients as features are used in diverse applications including visual explanations (Selvaraju et al., 2017; Prabhushankar et al., 2020; Prabhushankar and AlRegib, 2021b), adversarial attacks (Goodfellow et al., 2014), anomaly detection (Kwon et al., 2020), and image quality assessment (Kwon et al., 2019) among others. In this work, we use gradients as features to characterize data.

##### 2.3.2.1. MLP [H(·)]

Once *r*_*x*_ is obtained for all *N* classes, the surprisal feature is now analogous to *z*_*d*_ from Equation (5). However, *r*_*x*_ is of dimensionality ${ℜ}^{\left(N\times d_{L-1}\right)\times 1}$ since it is a concatenation of *N* gradients. To account for the larger dimension size, we classify *r*_*x*_ by training an MLP H(·) on top of *r*_*x*_ derived from training data. In this paper, we use a simple three layered MLP as H(·) with sigmoid activations. The exact structure of the MLP is dependent on *d*_*L*−1_ of the base *f*(·) network and is given in Table 1 for ResNets 18,34,50, and 101 (He et al., 2016) that are considered in Section 3.

**Table 1:** Structure of H(·) for different ResNet architectures as *f*(·).

**Network *f*(·)**	**Structure of H(·)—All layers separated by sigmoid**
ResNet-18, 34	640 × 300 − 300 × 100 − 100 × 10
ResNet-50, 101	2, 560 × 300 − 300 × 100 − 100 × 10

##### 2.3.2.2. Training H(·)

The concatenated *r*_*x*_ features for all training data are extracted and normalized. H(·) is trained on all training *r*_*x*_ using the same training procedure as the perception network *f*(·). H(·) is trained for 200 epochs with SGD optimizer and cross-entropy loss, momentum = 0.9, weight decay = 5*e*−4, and adaptive learning rates of 0.1, 0.02, 0.004 changed at epochs 60, 120, 160, respectively.

##### 2.3.2.3. Testing using *f*(·) and H(·)

During test time, the proposed framework operates in three steps. In step 1, as shown in Equation (13), the given image passes through the perception network to provide a coarse estimation ŷ. In step 2, the stochastic surprisal features *r*_*x*_ are extracted according to Equation (14) and concatenated. In step 3, *r*_*x*_ is normalized and passed through the MLP H(·) to obtain the final prediction ỹ. This is shown in Equation (15).


(13) $$\begin{array}{l} ŷ=\mathrm{argmax}f\left(x\right), \end{array}$$

(14) $$\begin{array}{l} r_{x}=\left[\left(\nabla_{W_{L}}\text{MSE}\left(ŷ,\delta_{i}^{i}\right)\right),\forall i\in \left[1,N\right]\right], \end{array}$$

(15) $$\begin{array}{l} ỹ=H\left(r_{x}\right), \end{array}$$

Note that we substituted $A_{d}$ in Equation (14) with the MSE formulation of action from Equation (10).

## 3. Results

### 3.1. Image quality assessment

We report the results of the our proposed method in comparison with commonly cited methods in this section. We first discuss the datasets used for comparison as well as the evaluation metrics. We finally show the results in Table 2 and discuss these results.

**Table 2:** Overall performance of image quality estimators.

	**PSNR**	**SSIM**	**MS**	**CW**	**IW**	**SR**	**FSIM**	**FSIMc**	**BRIS**	**BIQI**	**BLII**	**Per**	**CSV**	**UNI**	**COHER**	**SUM**	**Proposed**
**Databases**	**HA**		**SSIM**	**SSIM**	**SSIM**	**SIM**			**QUE**		**NDS2**	**SIM**		**QUE**	**ENSI**	**MER**	
**Outlier ratio (OR**, ↓**)**																	
MULTI	0.013	0.016	0.013	0.093	0.013	**0.000**	0.018	0.016	0.067	0.024	0.078	0.004	**0.000**	**0.000**	0.031	**0.000**	**0.000**
TID13	**0.615**	0.734	0.743	0.856	0.701	0.632	0.742	0.728	0.851	0.856	0.852	0.655	0.687	0.640	0.833	**0.620**	**0.620**
**Root mean square error (RMSE**, ↓**)**																	
MULTI	11.320	11.024	11.275	18.862	10.049	**8.686**	10.866	10.794	15.058	12.744	17.419	9.898	9.895	9.258	14.806	8.212	**7.943**
TID13	0.652	0.762	0.702	1.207	0.688	**0.619**	0.710	0.687	1.100	1.108	1.092	0.643	0.647	0.615	1.049	0.630	**0.596**
DRv1	16.19	17.11	16.17	17.18	14.02	13.64	**12.98**	**13.24**	–	–	–	16.01	15.07	13.59	21.82	16.98	13.85
DRv2	16.47	16.42	15.76	17.48	14.04	13.17	**12.82**	**12.92**	–	–	–	16.23	15.35	13.19	21.57	17.59	13.24
**Pearson linear correlation coefficient (PLCC**, ↑**)**																	
MULTI	0.801	0.813	0.803	0.380	0.847	0.888	0.818	0.821	0.605	0.739	0.389	0.852	0.852	0.872	0.622	**0.901**	**0.908**
	−1	−1	−1	−1	−1	0	−1	−1	−1	−1	−1	−1	−1	−1	−1	0	
TID13	0.851	0.789	0.830	0.227	0.832	0.866	0.820	0.832	0.461	0.449	0.473	0.855	0.853	**0.869**	0.533	0.861	**0.877**
	−1	−1	−1	−1	−1	0	−1	−1	−1	−1	−1	−1	−1	0	−1	−1	
DRv1	0.731	0.693	0.732	0.586	0.800	0.819	**0.833**	**0.830**	–	–	–	0.738	0.738	0.820	0.432	0.698	0.800
	−1	−1	−1	−1	0	1	1	1	–	–	–	−1	−1	1	−1	−1	
DRv2	0.709	0.702	0.738	0.521	0.799	0.826	**0.836**	**0.833**	–	–	–	0.720	0.720	0.825	0.417	0.658	0.815
	−1	−1	−1	−1	−1	0	1	1	–	–	–	−1	−1	0	−1	−1	
**Spearman's rank correlation coefficient (SRCC**, ↑**)**																	
MULTI	0.715	0.860	0.836	0.631	**0.884**	0.867	0.864	0.867	0.598	0.611	0.386	0.818	0.849	0.867	0.554	**0.884**	**0.887**
	−1	0	−1	−1	0	0	0	0	−1	−1	−1	−1	−1	0	−1	0	
TID13	0.847	0.742	0.786	0.563	0.778	0.807	0.802	0.851	0.414	0.393	0.396	0.854	0.846	**0.860**	0.649	0.856	**0.865**
	−1	−1	−1	−1	−1	−1	−1	−1	−1	−1	−1	0	−1	0	−1	0	
DRv1	0.739	0.702	0.738	0.760	0.798	0.807	**0.823**	**0.820**	–	–	–	0.742	0.769	0.810	0.518	0.706	0.807
	−1	−1	−1	−1	−1	0	1	1	–	–	–	−1	−1	0	−1	−1	
DRv2	0.720	0.705	0.738	0.755	0.795	0.809	**0.819**	**0.816**	–	–	–	0.727	0.755	0.813	0.525	0.672	**0.816**
	−1	−1	−1	−1	−1	−1	0	0	–	–	–	−1	−1	0	−1	−1	
**Kendall's rank correlation coefficient (KRCC**, ↑**)**																	
MULTI	0.532	0.669	0.644	0.457	**0.702**	0.678	0.673	0.677	0.420	0.440	0.268	0.624	0.655	0.679	0.399	0.698	**0.702**
	−1	0	0	−1	0	0	0	0	−1	−1	−1	−1	0	0	−1	0	
TID13	0.666	0.559	0.605	0.404	0.598	0.641	0.629	0.667	0.286	0.270	0.277	**0.678**	0.654	0.667	0.474	0.667	**0.677**
	0	−1	−1	−1	−1	−1	−1	0	−1	−1	−1	0	0	0	−1	0	
DRv1	0.534	0.505	0.537	0.559	0.597	0.609	**0.629**	**0.626**	–	–	–	0.537	0.563	0.609	0.357	0.503	0.605
	−1	−1	−1	−1	0	0	1	1	−	–	–	−1	−1	0	−1	−1	
DRv2	0.517	0.509	0.539	0.553	0.595	0.613	**0.626**	**0.623**	–	–	–	0.525	0.594	0.613	0.342	0.475	0.616
	−1	−1	−1	−1	−1	0	1	0	–	–	–	−1	−1	0	−1	−1	

The top 2 performing estimators in each row are bold faced and blue highlighted.

#### 3.1.1. Datasets

We compare our proposed quality estimation technique on three datasets—MULTI-LIVE (Jayaraman et al., 2012), TID2013 (Ponomarenko et al., 2015), and DR IQA (Athar and Wang, 2023). We choose MULTI-LIVE and TID2013 datasets for two reasons. Firstly, our proposed technique is a plug-in approach on top of an existing technique (Temel et al., 2016). Hence, it is imperative to compare against and show results on datasets that were used in Temel et al. (2016). Secondly, the two datasets provide access to seven categories of distortion among five levels. This is useful in comparison against the recognition experiments discussed in Section 3.2 which follows a similar setup. The complex distortions can either be a combination of multiple distortions such as distortions generated in the MULTI-LIVE dataset (Jayaraman et al., 2012) or the human visual system (HVS) specific peculiar distortions such as the ones presented in the TID2013 (Ponomarenko et al., 2015) dataset. A more challenging scenario is presented in DR IQA dataset, where the authors conjecture a degraded reference setting for image quality assessment. In this setting, pristine images are unavailable as a reference. Instead, singly distorted images are used as reference to construct IQA metrics for multiply distorted images. In Table 2, we provide results for DR IQA dataset as DRv1 and DRv2 based on the author's division of the dataset. Each of DRv1 and DRv2 have 31, 790 multiply distorted images and 1, 122 singly distorted images. Additionally, this dataset does not have *true* subjective quality scores from humans but is derived from a synthetic quality benchmark. This synthetic score uses existing Full Reference metrics for quality generation including some of comparisons in Table 2.

#### 3.1.2. Evaluation metrics

The performance is validated using outlier ratio (consistency), root mean square error (accuracy), Pearson correlation (linearity), Spearman correlation (rank), and Kendall correlation (rank). Arrows next to each metric in Table 2 indicate the desirability of a higher number (↑) or a lower number (↓). Statistical significance between correlation coefficients is measured with the formulations suggested in ITU-T Rec. P.1401 (ITU-T, 2012) and provided below each correlation coefficient. A 0 value corresponds to statistically similar performance, -1 means the method is statistically inferior to proposed method, and 1 indicates that the method is statistically superior to proposed method. Two best performing methods for each metric are highlighted.

#### 3.1.3. Results

We compare our proposed stochastic surprisal-based UNIQUE against other image quality estimators based only on handcrafted features and perception pipeline in Table 2. These compared full reference estimators include PSNR-HA (Ponomarenko et al., 2011), SSIM (Wang et al., 2004), MS-SSIM (Wang et al., 2003), CW-SSIM (Sampat et al., 2009), IW-SIM (Wang and Li, 2011), SR-SIM (Zhang and Li, 2012), FSIM (Zhang et al., 2011), FSIMc (Zhang et al., 2011), PerSIM (Temel and AlRegib, 2015), CSV (Temel and AlRegib, 2016b), UNIQUE (Temel et al., 2016). We also compare against no reference metrics including BRISQUE (Mittal et al., 2012), BIQI (Moorthy and Bovik, 2010), and BLIINDS2 (Saad et al., 2012). All these techniques were also compared against the base UNIQUE algorithm in Temel et al. (2016). In addition to these, we compare against new estimators including COHERENSI (Temel and AlRegib, 2019) and SUMMER (Temel and AlRegib, 2019). SUMMER beats UNIQUE among six of the 10 categories. Note that we do not show results for BRISQUE, BIQI, and BLIINDS2 for DR IQA dataset since NR methods, that are generally trained on singly distorted images, exhibit a large performance gap on multiply distorted images (Athar and Wang, 2023).

The proposed stochastic surprisal-based method plugs on top of UNIQUE and its results are provided under the last column in Table 2. It is always in the top two methods for MULTI-LIVE and TID2013 datasets in all evaluation metrics. In particular, the proposed method achieves the best performance for all the categories except in OR and KRCC in TID2013 dataset. UNIQUE, by itself, does not achieve the best performance for any of the metrics in MULTI dataset. However, the same network using the proposed gradient features significantly improves the performance and achieves the best performance on all metrics. For instance, UNIQUE is the third best performing method in MULTI dataset in terms of RMSE, PLCC, SRCC, and KRCC. However, the action-based features improve the performance for those metrics by 1.315, 0.036, 0.020, and 0.023, respectively and achieve the best performance for all metrics. This further reinforces the plug-in capability of the proposed method during inference. On DR IQA dataset, FSIM and FSIMc perform the best across all categories. The authors in Athar and Wang (2023) used FSIMc to construct DR IQA models. However, the proposed algorithm remains competitive among all evaluation metrics. The results are statistically significant in 53 of the 78 compared metrics across both DRv1 and DRv2. Note that a number of these compared FR-IQA metrics have been utilized to construct the synthetic ground truth quality scores.

### 3.2. Robust classification

Neural networks are sensitive to distortions in test that the network was not privy to during training (Temel et al., 2017, 2018; Hendrycks and Dietterich, 2019). These distortions include image acquisition errors, environmental conditions during acquisition, transmission, and storage errors among others. CIFAR-10C (Hendrycks and Dietterich, 2019) dataset consists of 19 real world distortions each of which has five levels of degradation that distort the 10, 000 images in CIFAR-10 testset. Neural networks that use perception-only mechanics suffer performance accuracy drops on CIFAR-10C. Current techniques that alleviate the drop in perception-only accuracy require additional training data. The authors in Vasiljevic et al. (2016) show that finetuning or retraining networks using distorted images increases the performance of classification under the same distortion. However, performance between different distortions is not generalized well. For instance, training on gaussian blurred images does not guarantee a performance increase in motion blur images (Geirhos et al., 2018b). Other proposed methods include training on style-transferred images (Geirhos et al., 2018a), training on adversarial images (Hendrycks and Dietterich, 2019), training on simulated noisy virtual images (Temel et al., 2017), and self-supervised methods like SimCLR (Chen et al., 2020) that train by augmenting distortions. Augmix (Hendrycks et al., 2019) creates multiple chains of augmentations to train the base network. All these works require additional training data. Our proposed stochastic surprisal-based technique is a plug-in on top of any existing method that increases the base network's robustness to distortions without any need for new data.

#### 3.2.1. Experimental setup and dataset

We use CIFAR-10C (Hendrycks and Dietterich, 2019) as our dataset of choice with all its 95 distortions and degradation levels. ResNet-18,34,50, and 101 (He et al., 2016) architectures are used as the base *f*(·) perception-only networks. These are trained from scratch on CIFAR-10 dataset. Following the terminologies established in Section 2, X is the training set of CIFAR-10 and $X^{\prime }$ are the 19 distorted domains in which the testing set of CIFAR-10C reside. Each of the 19 corruptions have five levels of distortions. Higher the level, higher is the distortion. The distortions include blur characteristics like gaussian blur, zoom blur, glass blur, and environmental distortions like rain, snow, fog, haze among others.

#### 3.2.2. Comparison against existing state of the art methods

In Table 3, we compare the Top-1 accuracy between perception-only inference and our proposed stochastic surprisal-based inference. All the state-of-the-art techniques require additional training data—noisy images (Vasiljevic et al., 2016), adversarial images (Hendrycks and Dietterich, 2019), self-supervision SimCLR augmentations (Chen et al., 2020), and augmentation chains (Hendrycks et al., 2019). We term these perception-only techniques as *f*′(·) and we actively infer on top of them. For all *f*′(·) other than Augmix, the base network is a ResNet-18. For Augmix, we use WideResNet architecture following the authors in Hendrycks et al. (2019). Another commonly used robustness technique is to pre-process the noisy images to denoise them. Denoising 19 distortions is, however, not a viable strategy assuming that the characteristics of the distortions are unknown. We use Non-Local Means (Buades et al., 2011) denoising and the results obtained are lower than the perception-only accuracy by almost 3%. However, the proposed technique on this model increases the results by 3.84%. We create untargeted adversarial images using FGSM attack (Goodfellow et al., 2014) and use them to train a ResNet-18 architecture. In the experimental setup of augmenting noise (Vasiljevic et al., 2016), we augment the training data of CIFAR-10 with six distortions provided by Temel et al. (2018) to randomly distort 500 CIFAR-10 training images to train *f*′(·). For all techniques, the proposed technique plugs on top of *f*′(·) and increases the accuracy to create robust networks. Note that in all the perception-only methods in Table 3, we do not use the augmented data to train H(·). The gain obtained is by creating actions on only the undistorted data. Even when the augmented network *f*′(·) gains on non-augmented *f*(·), the proposed technique plugs on top of *f*′(·) to provide additional gains.

**Table 3:** Stochastic surprisal-based plug-in on top of existing robustness techniques.

**Methods**		**Accuracy (%)**
ResNet-18	Perception-only	67.89
	Proposed	**71.4**
Denoising	Perception-only	65.02
	Proposed	**68.86**
Adversarial train (Hendrycks and Dietterich, 2019)	Perception-only	68.02
	Proposed	**70.86**
SimCLR (Chen et al., 2020)	Perception-only	70.28
	Proposed	**73.32**
Augment noise (Vasiljevic et al., 2016)	Perception-only	76.86
	Proposed	**77.98**
Augmix (Hendrycks et al., 2019)	Perception-only	89.85
	Proposed	**89.89**

The higher accuracy between the perception-only and the proposed technique for every method is highlighted in bold.

#### 3.2.3. Analyzing distortion-wise accuracy gains

**Figure 4:**
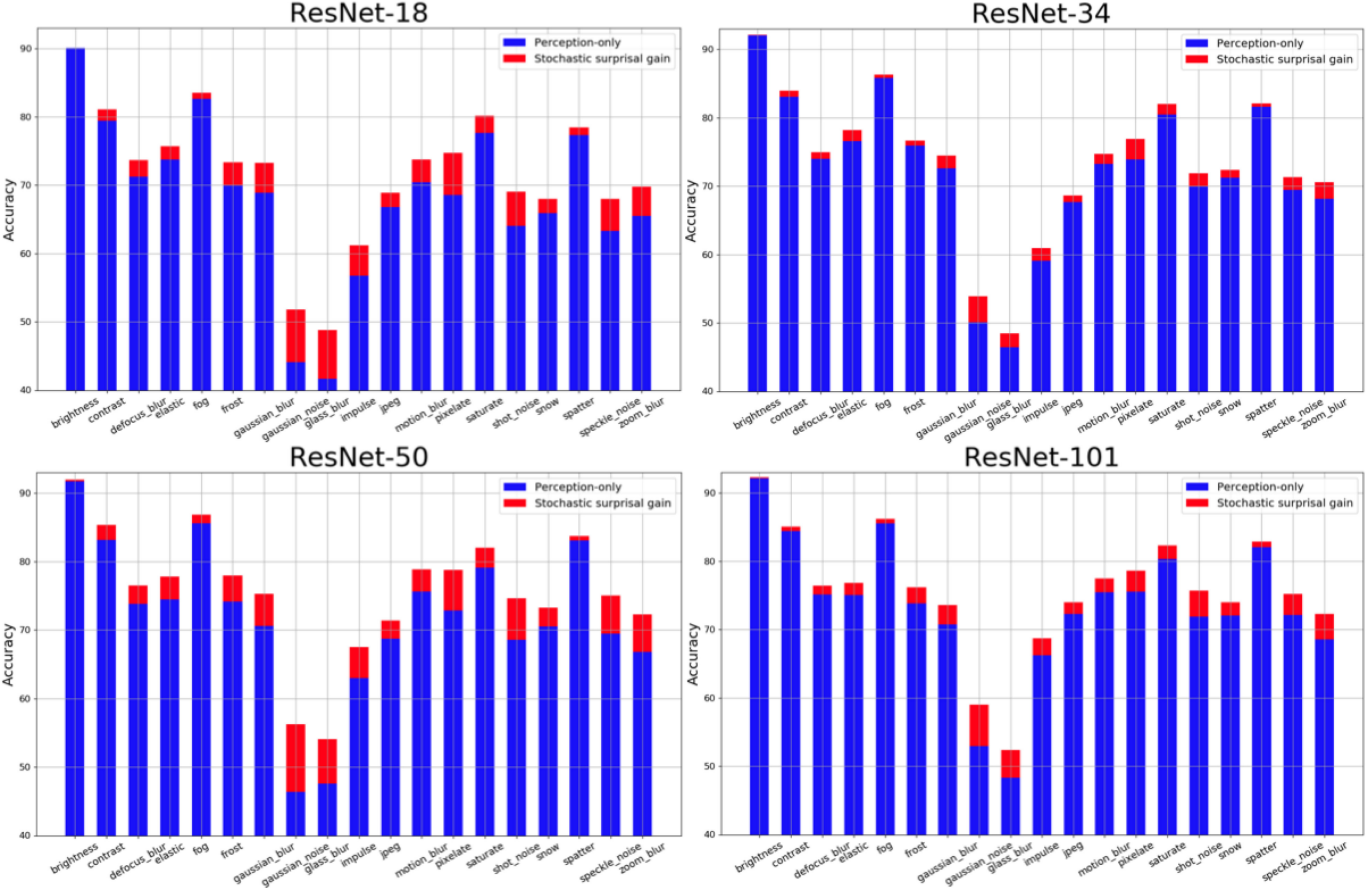
Visualization of accuracy gains (in red) of using the proposed stochastic surprisal-based inference over perception-only inference (in red) on CIFAR-10C dataset (Hendrycks and Dietterich, 2019) for four networks across 19 distortions.

The results of all four ResNet architectures for each of the 19 distortions is shown in Figure 4. X-Axis in each plot shows 19 distortions averaged over all 5 distortion levels. Y-Axis shows Top-1 accuracy. The bars in blue show perception-only inference results and the red region in each bar represents the performance gain obtained by stochastic surprisal-based inference. There is an increase in performance across distortions and networks. In 9 of the 19 distortions, the proposed method averages 4% more than its perception-only counterpart. These include gaussian blur, gaussian noise, glass blur, impulse noise, motion blur, pixelate, shot noise, speckle noise, and zoom blur. The highest increase is 8.22% for glass blur. In 2 of the distortions, brightness and saturate, the results increase by < 0.4% averaged over all levels. This is because of the statistics that the distortions affect. Distortions can change either the local or global statistics within images. Distortions like saturate, brightness, contrast, fog, and frost change the low level or global statistics in the image domain. Neural networks are actively trained to ignore such changes so that their effects are not propagated beyond the first few layers. Hence, gradients derived from the final fully connected layer do not capture the necessary changes required within *f*(·) to compensate for these distortions. Therefore, both the proposed and perception-only inference follow each other closely in distortions like brightness and saturate.

#### 3.2.4. Level-wise recognition on CIFAR-10C

**Figure 5:**
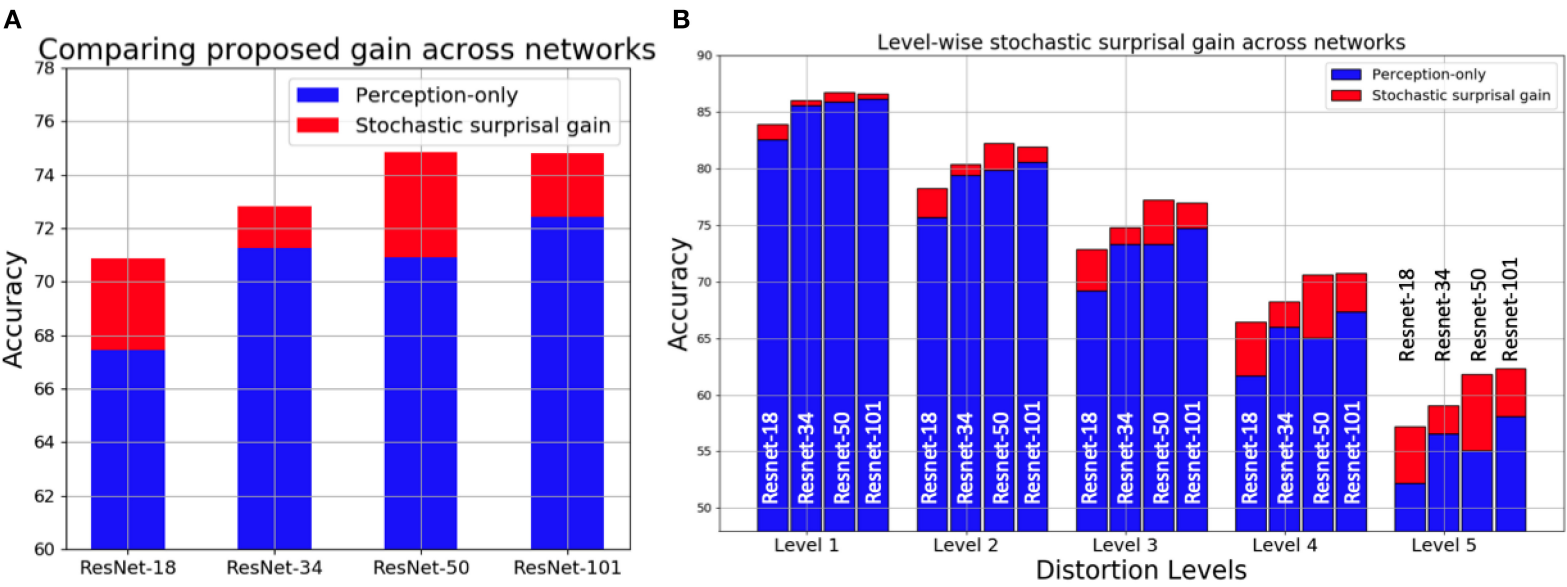
Visualization of accuracy gains (in red) of using the proposed stochastic surprisal-based inference over perception-only inference (in blue) on CIFAR-10C dataset (Hendrycks and Dietterich, 2019) for four networks **(A)** averaged across 19 distortions and 5 levels **(B)** shown across 5 levels of distortion.

In Figure 5B, the proposed performance gains for the four networks are categorized based on the distortion levels. All 19 categories of distortion on CIFAR-10C are averaged for each level and their respective perception-only accuracy and stochastic surprisal-based gains are shown. Note that the levels are progressively more distorted. Hence, level 1 distribution $X^{\prime }$ is similar to the training distribution X when compared to level 5 distributions. As the distortion level increases, the proposed method's accuracy gains also increase. This is because, with a larger distributional shift, more characteristic is the action required w.r.t. the network parameters. In Figure 5A, we show the distortion-wise and level-wise accuracy gains for each network. Note that, a stochastic surprisal-based ResNet-18 performs similarly to a perception-only ResNet-50.

## 4. Discussion

We conclude this paper by considering the terminology of stochastic surprisal as well as some of the broader implications of the proposed technique. These include the abductive reasoning module and expectancy-mismatch hypothesis in cognitive science.

### 4.1. Choice of the terminology of stochastic surprisal

We motivate the terminology of *stochastic surprisal* in two ways:

- As an analogy to *gradient descent* and *stochastic gradient descent*: Gradient descent requires the gradients from the all available training data to update the weights. However, since this is computationally infeasible for large neural networks, stochastic gradient descent allows using a single training datapoint to estimate gradients, repeated across all data. In *stochastic surprisal*, we use the single data point, available at inference, under all allowable actions to estimate surprisal.
- Meaning of stochastic: The word stochastic implies some randomness within the setting. This randomness is derived from the possible set of all actions. In discriminative networks in Equation (10), *a*_*i*_, *i* ∈ [1, *N*] is the set of all possible actions with *N* being the number of trained classes. This suggests that we allow a datapoint to be any available class, all of which are equally likely. Similarly, in generative networks in Equation (9), we add random perturbations at the output of the autoencoder. Hence, there is an inherent randomness within the actions that allow for the usage of the word *stochastic*.

### 4.2. Abductive reasoning

**Figure 6:**
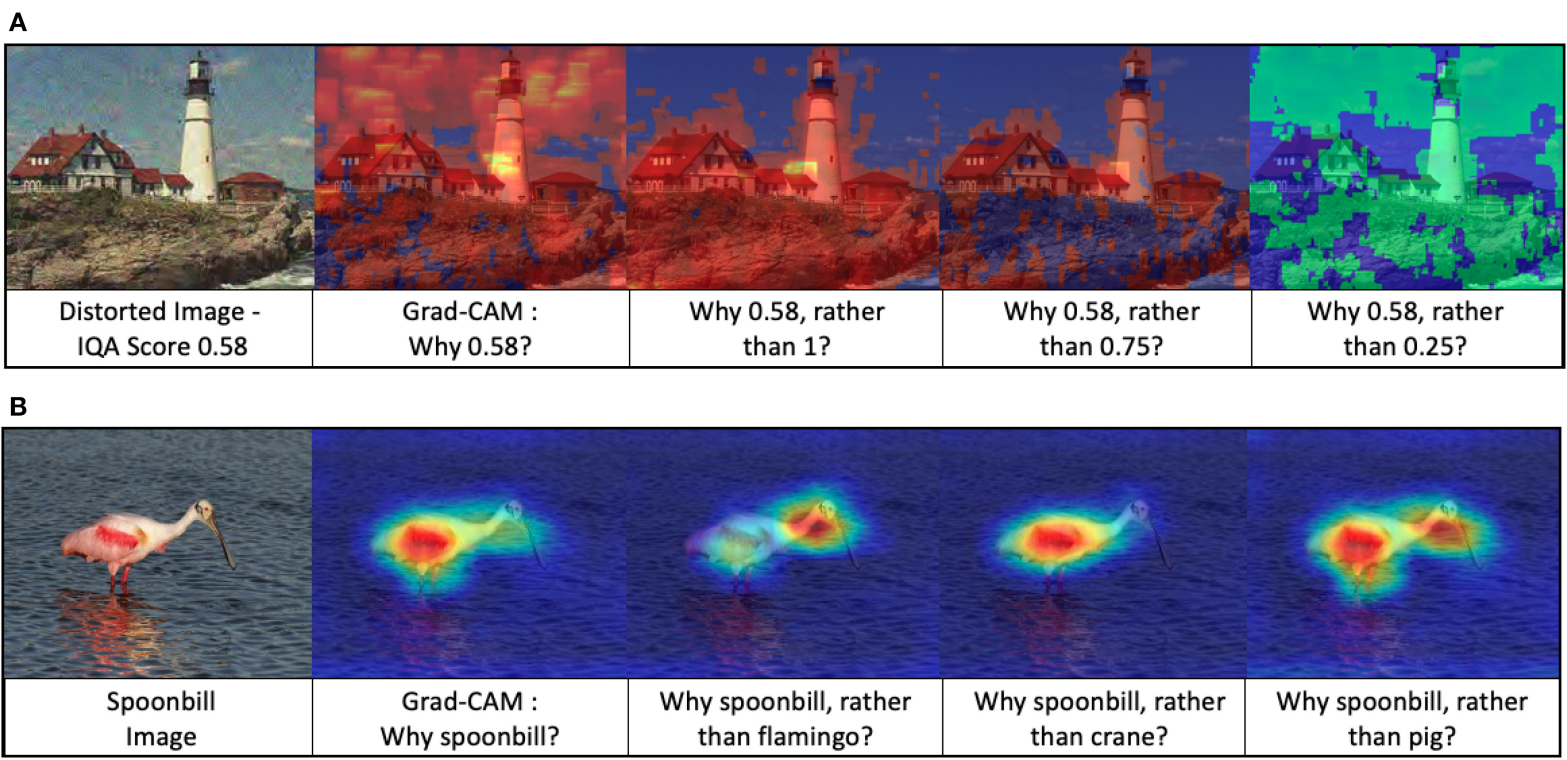
Stochastic surprisal answers contrastive questions. **(A)** Grad-CAM and contrastive explanations for the application of image quality assessment and **(B)** Grad-CAM and contrastive explanations for the application of recognition. The highlighted regions in each image provide a visual explanation to the question beneath it. While Grad-CAM (Selvaraju et al., 2017) shows all the perceived regions in the image, the stochastic surprisal provides fine-grained answers to contrastive questions. Best viewed in color.

The free energy principle postulates that the brain encodes a Bayesian recognition density that predicts sensory data based upon some hypotheses about their causes. This mode of inference is called inference to the best explanation. The underlying reasoning model is abductive reasoning. Abductive reasoning was introduced by the philosopher Peirce (1931), who saw abduction as a reasoning process from effect to cause (Paul, 1993). An abductive reasoning framework creates a hypothesis and tests its validity without considering the cause. A hypothesis can be considered as an answer to one of the three following questions: a causal “*Why P?”* question, a counterfactual “*What if?”* question, and a contrastive “*Why P, rather than Q?”* question (AlRegib and Prabhushankar, 2022). Here *P* is the prediction and *Q* is any contrast class. The action considered in this paper is the latter contrastive question of the form “*Why P, rather than Q?”*. Stochastic surprisal measures the answer to this question. We explore this further in Prabhushankar et al. (2020) and AlRegib and Prabhushankar (2022). We borrow the visualization procedure from Prabhushankar et al. (2020) to visually analyze stochastic surprise in the applications of IQA and recognition in Figure 6. We do so to illustrate the broader impact of action at inference time. As in Section 2.3.1.2, we use stochastic surprisal as a plug-in approach.

For IQA visualizations, we use a trained full-reference metric DIQaM-FR Bosse et al. (2017) as our perception model. In Figure 6A, the pretrained network from Bosse et al. (2017) provides a quality score of 0.58 to the distorted lighthouse image. Here 0.58 acts as *P* in the contrastive question. We use MSE loss function as $A_{d}$ and a real number *Q* ∈ [0, 1] to calculate stochastic surprisal. Contrastive explanations of *Q* values including 0.25, 0.75, and1 along with Grad-CAM results are shown in Figure 6A. Grad-CAM highlights the entire image indicating that the network estimates the quality based on the whole image. While this builds trust in the network, it does not help us understand the network decision. The stochastic surprisal, however, provides fine-grained explanations. Consider the contrastive questions asking why the quality is neither 1nor0.75. The network estimates this to be primarily due to distortions concentrating in the foreground portion of the image. This explanation is inline with previous works in IQA that posit that distortions in the more salient foreground or edge features cause a larger drop in perceptual quality than that in color or background (Chandler, 2013; Prabhushankar et al., 2017b). When the contrastive question asks why the prediction is not 0.25, the network highlights the sky indicating its good quality for a higher score of 0.58.

Figure 6B shows the contrastive questions answered by the stochastic surprisal for the application of recognition. Given an image of a spoonbill from ImageNet dataset (Deng et al., 2009), a VGG-16 network highlights the body, feathers, legs and beak of the bird in the Grad-CAM (Selvaraju et al., 2017) explanation. Consider a more fine grained contrastive question regarding the difference between a spoonbill and flamingo. The stochastic surprisal highlights regions in the neck of the spoonbill indicating that the contrast between the input spoonbill image and the network's notion of a flamingo lies in the spoonbill's lack of S-shaped neck. Similarly, the contrast between a spoonbill and a crane is in the color of the spoonbill's feathers. The contrast between a pig and a spoonbill is in the shape of neck and legs in the spoonbill which is emphasized. All these visualizations serve to illustrate the stochastic nature of the proposed method. It is stochastic in the sense that it individually depends on the network, the data, as well as the action. In this case, the action of not predicting a flamingo has a different explanation compared to the action of not predicting a pig.

### 4.3. Expectancy-mismatch

The expectancy-mismatch hypothesis in cognitive science is a way to quantify and analyze human attention. According to this hypothesis, human attention mechanism suppresses expected messages and focuses on the unexpected ones (Horstmann, 2002; Summerfield and Egner, 2009; Becker and Horstmann, 2011; Krebs et al., 2012; Horstmann et al., 2016; Sun et al., 2020). Becker and Horstmann (2011) shows that a message which is unexpected, captures human attention. Then, the human visual system establishes whether the input matches the observers' expectation. If they are conflicting, error neurons in the human brain encode the prediction error and pass the error message back to the representational neurons. The proposed method uses gradients with respect to the network parameters to measure an action. In both the generative and discriminative networks, this action takes the form of a change in the output thereby creating a mismatch with the network's expected result. Hence, the proposed method can act as a framework for exploring expectancy-mismatch in future works.

### 4.4. Related learning paradigms

The proposed stochastic surprisal decomposes the decision making and training process of a neural network into perception and action phases. A number of other machine learning paradigms including continual and lifelong learning (Parisi et al., 2019), online learning (Hoi et al., 2021), and introspective learning (Prabhushankar and AlRegib, 2022) also have multiple stages. Online learning assumes an exploration and exploitation stage in a neural network's training process. Hence, the differentiation in the training stages is based on time rather than the proposed action. Continual and lifelong learning is a research paradigm that tackles the topic of catastrophic forgetting when a neural network is trained to perform multiple tasks. Introspective learning conjectures reasons in the form of counterfactual or contrastive questions in its two stages to make predictions. Hence, while there are multiple machine learning paradigms that conjecture decomposition of neural network's training and decision processes, the proposed framework that is based on the FEP is unique in its decomposition. The field of active learning (Benkert et al., 2022; Logan et al., 2022) involves actions within the training and decision making processes. However, active learning requires actions from the users while the considered actions in the proposed methodology are with respect to the neural network.

## 5. Conclusion

In this paper, we examine supervised learning from the perspective of Free Energy Principle. The learning process of both generative and discriminative models can be decomposed into divergence and surprisal measures. Surprisal is introduced in generative models *via* regularization and constraints that allow a generative aspect to their functionality. While this complicates the action itself, the set of possible actions is still limited. Discriminative networks follow the traditional route of free energy minimization by defining surprisal in terms of recognition entropy and minimizing it. This allows the action itself to be a simple fidelity-based reconstruction error. However, in discriminative networks, there are *N* set of possible actions, *N* being the number of classes in the recognition density. We account for both these peculiarities in defining our action space. We use a fidelity-based MSE loss for both generative and discriminative networks. In addition, generative networks are reinforced with KL-divergence based elastic net regularization, and in discriminative networks we backpropagate *N* possible actions. We measure this scalar action quantity in terms of a vector quantity called stochastic surprisal that is a function of the network parameters and an individual data point rather than a distribution. We use stochastic surprisal to assess distortions in image quality assessment and disregard distortions in robust recognition. We then discuss the implications of stochastic surprisal in other areas of cognitive science including abductive reasoning and expectancy-mismatch. A computational bottleneck within the framework is the consideration of all *N* possible actions to estimate the surprisal feature *r*_*x*_. *r*_*x*_ scales linearly with *N* thereby becoming prohibitive on datasets with a large number of classes. Selecting only a subset of the most likely actions is one plausible solution to the challenge of scalability.

## Data availability statement

Publicly available datasets were analyzed in this study. This data can be found at: CIFAR-10C: https://zenodo.org/record/2535967; CIFAR-10: https://www.cs.toronto.edu/~kriz/cifar.html; TID2013: https://www.ponomarenko.info/tid2013.htm; MULTI-LIVE: http://live.ece.utexas.edu/research/Quality/live_multidistortedimage.html.

## Author contributions

MP wrote the first draft of the manuscript. GA and MP had multiple rounds of revisions and approved the submitted version. All authors contributed to the article and approved the submitted version.
